# Transvenous extraction failure of a recently implanted active fixation coronary sinus lead: Good or bad times?

**DOI:** 10.1016/j.hrcr.2024.01.009

**Published:** 2024-01-26

**Authors:** Andrea Di Cori, Lorenzo Pistelli, Matteo Parollo, Attilio Lepone, Mario Giannotti Santoro, Giulio Zucchelli

**Affiliations:** ∗ Second Division of Cardiology, Cardio-Thoracic and Vascular Department, Pisa University Hospital, Pisa, Italy; † Department of Clinical and Experimental Medicine, University of Messina, Messina, Italy; ‡ Department of Translational Research and New Technologies in Medicine and Surgery, University of Pisa, Pisa, Italy

**Keywords:** Cardiac resynchronization therapy, Coronary sinus pacing lead, Active fixation lead, Transvenous lead extraction

**Key Teaching Points**

• Extraction of the active coronary sinus fixation lead 4798 Attain Stability (Medtronic, Minneapolis, MN) has proven to be potentially challenging, even following a dwelling period of less than 1 year.
• Fibrosis or thrombus formation may contribute to the difficulty in removal, despite counterclockwise rotation of the fixed coaxial helix and dilation, even after a short dwell period and particularly in patients exhibiting a strong fibrotic reaction.
• Difficulties in extraction of active fixation coronary sinus lead underscore the importance of judicious use of these leads. They should be reserved for highly selective cases where the likelihood of device infection or the risk of long-term complications is relatively low, and no other viable options (such as conductive system pacing) are reasonable.
• In recognition of the importance of a complete lead removal, and the complexities associated with the extractability of coronary sinus active fixation leads, these procedures should be performed by expert operators in specialized centers, where modern and sophisticated approaches are available options.

## Introduction

Cardiac resynchronization therapy (CRT) has been shown to reduce morbidity and mortality in select patients with heart failure with reduced left ventricle ejection fraction (LVEF); however, in about 10% of patients, CRT fails owing to coronary sinus (CS) lead dislodgment.^1^, ^2^, ^3^, ^4^ Over the past 15 years, active fixation CS leads have been developed to address the need for greater stability of the CS lead.^3^ Attain StarFix 4195 (Medtronic, Minneapolis, MN), a 5F unipolar steroid-eluting CS lead with an extendable lobe for lead active fixation, demonstrated a significant improvement in dislodgment rates, but with a lower extractability rate, even after a short dwelling period.^3^^,^^5^ More recently, the Attain Stability 4798 (Medtronic, Minneapolis, MN), a 4F dual-electrode lead that actively fixes to the CS wall through an exposed side helix, has shown an improved CS lead implant success rate, of up to 97%.^6^ Limited data are available regarding the extractability of this novel CS lead. However, assessing the feasibility of the Attain Stability lead is crucial, particularly considering the steady increase in cardiovascular implantable electronic device implants and, consequently, removal over the years.^7^

We present the case of a 56-year-old woman who experienced an unsuccessful transvenous removal of a 4798 Medtronic Attain Stability after a very short (<1 year) implantation time.

## Case report

A 56-year-old woman with a dilated cardiomyopathy was referred to our center because of cardiovascular implantable electronic device local infection with indication to transvenous removal. Only 1 year earlier, on May 2022, she had undergone implantation of an implantable cardiac defibrillator with resynchronization therapy (Entrant HF CRT-D; Abbott, Chicago, IL) in primary prevention for a diagnosis of symptomatic severely reduced LVEF (EF 20%) with a new-onset left bundle branch block with 180 ms QRS duration.

**Figure 1:**
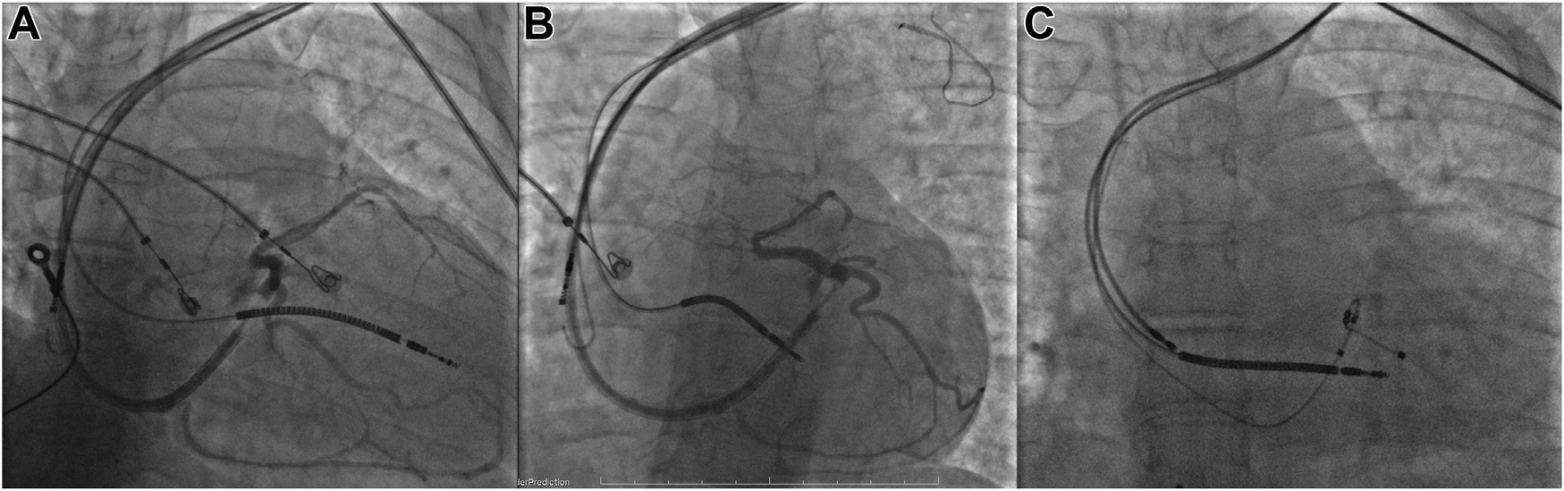
Previous implantation of implantable cardiac defibrillator with resynchronization therapy. **A, B:** Coronary sinus angiography. **C:** Placement of the Attain Stability (Medtronic, Minneapolis, MN) lead in the coronary sinus.

The right ventricular active-fixation defibrillating lead (Durata™ 7122Q 60 cm; Abbott, Chicago, IL) was placed at the apical low septum level through the left cephalic vein, while the atrial active fixation lead (Tendril™ STS 2088TC 52 cm; Abbott, Chicago, IL) and the CS lead were introduced via the left subclavian vein. Placement of the CS lead was remarkably challenging owing to unfavorable anatomy of the CS branches. After many attempts with a standard passive fixation lead, the only suitable kinked lateral branch one was approached (Figure 1) with an active fixation lead (Attain Stability 4798, 78 cm; Medtronic, Minneapolis, MN). According to the optimal QRS shortening result, clinical and volumetric CRT response were excellent, with an increase in LVEF up to 46%.

In July 2023, the patient was admitted to our electrophysiology unit for a pocket infection without fever. Blood tests indicated a mildly elevated white blood cell count with neutrophilia (white blood cells 14,000/μL, neutrophils 12,600/μL) and increased levels of C-reactive protein (2.8 mg/dL). Blood culture responses were negative. Transthoracic and intracardiac echo excluded lead-related endocarditis. Empiric antibiotic treatment was initiated with amoxicillin/clavulanate before extraction and was lated changed to daptomycin.

**Figure 2:**
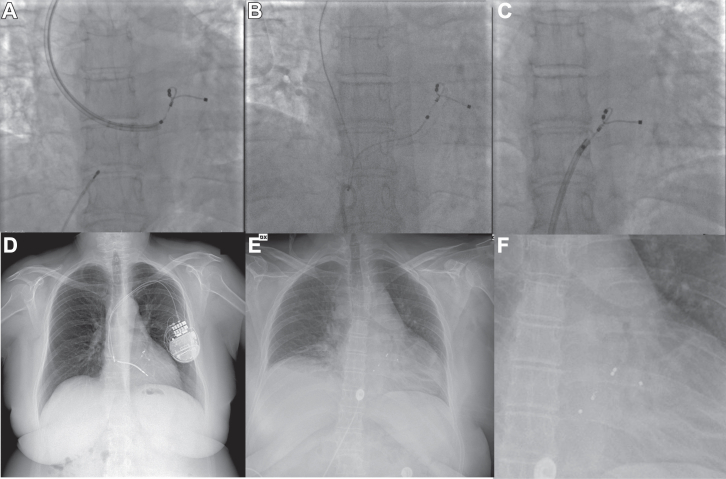
Attain Stability lead (Medtronic, Minneapolis, MN) extraction procedure. **A:** Mechanical dilation (MD) of adherence at the coronary sinus (CS) ostium through subclavian venous approach with polypropylene sheath. B: Externalization of the Attain Stability from the venous entry site to the right venous femoral access. **C:** MD of adherence at the CS ostium through right venous femoral access with the Agilis introducer (Abbott, Chicago, IL). **D:** Chest radiography before extraction. **E:** Chest radiography after extraction. **F:** Chest radiography after extraction with focus on the abandoned tip of the CS lead.

The extraction procedure was carried out under deep sedation and local analgesia, with invasive arterial blood pressure monitoring through right femoral artery access. Upon pocket opening, purulent material was drained. Owing to fibrotic adherences in the subclavian vein and at the junction between the subclavian and innominate vein, manual traction was ineffective for all 3 leads. Mechanical dilation using telescoping polypropylene sheaths (Byrd Sheaths; Cook Medical, Bloomington, IN) was necessary and allowed a successful extraction of the atrial and right ventricular leads. Through the left superior venous entry site, the CS lead was freed from adherences up to the CS ostium but required a crossover to an additional transvenous femoral approach (Supplemental Videos 1 and 2). Through the right femoral vein and under intracardiac echocardiography surveillance, despite the use of additional tools, including a deflectable long introducer (Agilis 12F; Abbott, Chicago, IL) deep inside the distal CS, a complete lead removal proved impossible, with a final rupture at the level of the proximal pole and the need for tip abandonment (Figure 2). No major or minor complications occurred during or after the procedure.

**Figure 3:**
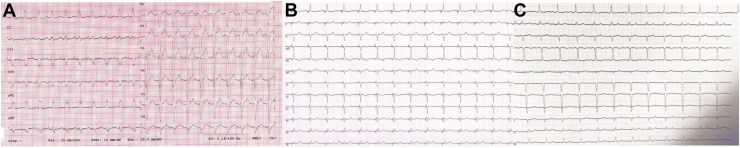
Electrocardiograms. **A:** Before implantation of implantable cardiac defibrillator with resynchronization therapy (CRT-D). **B:** During biventricular pacing. **C:** Resolved left bundle branch block after CRT-D removal.

Cultures from the extracted leads were all negative, whereas the pocket's purulent material tested positive for methicillin-resistant *Staphylococcus epidermidis*. Appropriate intravenous antimicrobial therapy was initiated and continued for 1 month based on antibiotic susceptibility testing. Subsequent echocardiographic assessment and electrocardiogram performed a few days after the procedure revealed a stable LVEF of 38%, with regression of the previous left bundle branch block (Figure 3). Consequently, the decision to reimplant a CRT-D was postponed to a subsequent follow-up reevaluation. After 3 months, the patient remained stable, without signs of infective clinical recurrences.

## Discussion

Considering the limited fixation mechanisms of most CS leads and the low propensity for fibrosis in CS and its tributary branches, most passive CS fixation leads can be extracted using manual traction alone, especially when the removal is done shortly after implantation.^8^ However, over a longer dwell time, adhesions may develop, necessitating proximal mechanical dilation in approximately one-third of cases.^2^^,^^8^ A recent report by Hayashi and colleagues^9^ showed that higher dwelling time and the CS lead as the first one to be approached during the procedure were independent predictors for incomplete CS lead extraction.

The novel 4798 Attain Stability lead (Medtronic) has demonstrated promising outcomes in terms of lead dislodgment rates. However, data on the feasibility of lead extraction are still lacking.^6^ A study by Adler and colleagues^10^ involving 12 sheep suggested that Attain Stability leads could be safely and easily extracted even up to 2 years postimplant. Nonetheless, no human studies were published on the extraction profile of Attain Stability leads.

Our case demonstrates for the first time potential challenges associated with transvenous extraction of the Attain Stability lead, even after a relatively short dwell time (1 year). Despite the design of a fixed coaxial helix, tissue retraction with counterclockwise rotation and/or dilation proved impossible. Fibrosis and/or thrombus formation are likely to account for the removal failure, and the fact that both atrial and ventricular leads needed mechanical dilation despite a 1-year dwelling time further suggests the patient’s strong fibrotic reaction. Moreover, we cannot exclude that the specific location of the active fixation mechanism (in the main CS, proximal to the target vessel) may have played a role. In the past, similar problems were observed with the StarFix lead (Medtronic), also immediately after implantation. Although the poor StarFix extraction profile was explained as the result of an inappropriate fixation mechanism, including the presence of a suboptimal push-lobes mechanism, the present case emphasizes once again the limitations of an active fixation strategy in the CS.^2^ The tip of the lead was left abandoned in the CS owing to the robust adhesion between the screw and the vessel wall, making the lead more prone to breakage than to allowing the screw to be successfully removed.^5^

Based on our experience, even though the majority of Attain leads can be easily removed, at least after a short dwelling time, a challenging procedure with possible failure or complications can not be excluded. It seems wise to consider active fixation CS leads only in extremely select cases, when lead stability cannot be achieved despite multiple attempts with standard leads and also after discussing the option of conduction system pacing. Furthermore, active fixation leads should be avoided in individuals at a higher risk of device infection (eg, chronic kidney disease or diabetes) and in younger patients, where the risk of long-term complication is incremental.

Finally, we cannot exclude that the removal of an active fixation CS lead should be conducted in specialized centers by operators with significant expertise in this type of procedure, considering the crucial role of complete system removal in case of device infection and potential risks related to abandoned material. Promising new approaches to CS lead extraction, such as the one recently described by Isonaga and colleagues^11^ involving a soft-tip guiding catheter, or hybrid approaches may represent viable options.^12^

## Conclusion

Similarly to the Attain StarFix active fixation lead, in our case Attain Stability has demonstrated resistance to transvenous extraction, even after a relatively short period of dwelling time (1 year). Further cases and, possibly, in-human studies are needed to accurately determine the feasibility of this lead extraction. However, based on our experience, it seems prudent to employ this type of lead in select cases that are at high risk of dislodgment and at low risk of future extraction. When necessary, the extraction of the Attain Stability lead should be conducted by highly skilled operators and in specialized centers, even if a brief period of time has elapsed after implant.

## Disclosures

The authors have no disclosures. The patient’s written informed consent was obtained.

## Appendix Supplementary Data

Video 1 CS lead crossing over from the left subclavian vein to the right femoral vein utilizing an Agilis 12 Fr introducer (Abbott, Chicago, IL, USA). Crossover procedure was performed after liberating the lead from adhesions up to the ostium of the coronary sinus through mechanical dilation. **CS:** coronary sinus.

Video 2 (Unsuccessful) Attempt to free the tip of the CS lead from adhesions deep inside the CS using an Agilis deflectable introducer (12 Fr, Abbott, Chicago, IL, USA) via a right femoral vein access. **CS:** coronary sinus.
